# Embolization of the middle meningeal artery vs. second surgery—treatment response and volume course of recurrent chronic subdural hematomas

**DOI:** 10.1007/s00701-023-05621-7

**Published:** 2023-05-29

**Authors:** Adrian Liebert, Heinz Voit-Höhne, Leonard Ritter, Thomas Eibl, Alexander Hammer, Michael Städt, Florian Eff, Markus Holtmannspötter, Hans-Herbert Steiner

**Affiliations:** 1 Department of Neurosurgery, Paracelsus Medical University, Breslauer Straße 201, 90471 Nuremberg, Bavaria Germany; 2 Department of Neuroradiology, Paracelsus Medical University, Breslauer Straße 201, 90471 Nuremberg, Bavaria Germany; 3 Center for Spinal and Scoliosis Surgery, Malteser Waldkrankenhaus St. Marien, Erlangen, Bavaria Germany

**Keywords:** Chronic subdural hematoma, Recurrence, Embolization, Volume, Treatment response

## Abstract

**Background:**

Despite multiple studies on the embolization of the middle meningeal artery, there is limited data on the treatment response of recurrent chronic subdural hematomas (CSDH) to embolization and on the volume change.

**Methods:**

We retrospectively compared the treatment response and volume change of recurrent CSDHs in a conventional group (second surgery) with an embolization group (embolization as stand-alone treatment) during the time-period from August 2019 until June 2022. Different clinical and radiological factors were assessed. Treatment failure was defined as necessity of treatment for second recurrence. Hematoma volumes were determined in the initial CT scan before first surgery, after the first surgery, before retreatment as well as in an early (1 day–2 weeks) and in a late follow-up CT scan (2–8 weeks).

**Results:**

Fifty recurrent hematomas after initial surgery were treated either by second surgery (*n* = 27) or by embolization (*n* = 23). 8/27 (26,6%) surgically treated and 3/23 (13%) of the hematomas treated by embolization needed to be treated again. This leads to an efficacy in recurrent hematomas of 73,4% in surgically treated and of 87% in embolized hematomas (*p* = 0.189). In the conventional group, mean volume decreased significantly already in the first follow-up CT scan from 101.7 ml (SD 53.7) to 60.7 ml (SD 40.3) (*p* = 0.001) and dropped further in the later follow-up scan to 46.6 ml (SD 37.1) (*p* = 0.001). In the embolization group, the mean volume did decrease insignificantly from 75.1 ml (SD 27.3) to 68 ml (SD 31.4) in the first scan (*p* = 0.062). However, in the late scan significant volume reduction to 30.8 ml (SD 17.1) could be observed (*p* = 0.002).

**Conclusions:**

Embolization of the middle meningeal artery is an effective treatment option for recurrent CSDH. Patients with mild symptoms who can tolerate slow volume reduction are suitable for embolization, whereas patients with severe symptoms should be reserved for surgery.

## Introduction

The incidence of chronic subdural hematomas varies from 1 to 17,6 per 100 000 individuals per year and is expected to rise steadily over the next decades [2, 21, 22]. The main burden of this common neurosurgical disease is the high recurrence rate with up to 39% after surgery [16]. Multiple approaches have been tested to tackle this issue in the past. Conservative therapy, for instance with dexamethasone or statins, additional to surgery were studied and indicated lower recurrence rates than surgery alone [19, 24, 27]. During the past decade a novel treatment option, the embolization of the middle meningeal artery has been performed and analyzed in multiple studies [8]. Due to promising results in these trials with a lower recurrence rate than surgery alone and little complication rates, this treatment option is increasingly performed [3, 8].

The underlying concept of this treatment option is the devascularization of the new immature capillaries which have grown into the outer membrane of the subdural hematoma [5]. After a mild head trauma, an inflammatory and pro-angiogenetic process is triggered, which forms an outer and inner membrane. Immature capillaries grow into the outer membrane. These immature leaky capillaries are the source of ongoing tiny bleedings into the newly formed space between the membranes and are supplied by the middle meningeal artery [4]. The concept of the embolization of the middle meningeal artery is to stop these ongoing minor bleedings through devascularization and allow resorption of the hematoma volume without continuous bleeding [5].

Middle meningeal artery embolization has been performed mostly as adjunctive treatment to surgery, but also as primary and rescue treatment option in recurrent hematomas [8]. However, data on the response of recurrent hematomas to embolization procedure and second recurrence is limited [9, 13, 14, 18].

Furthermore, in most of the published studies, the hematoma volume is not measured, and the efficacy of this treatment is based on the need for rescue surgery or reduction of maximal width as treatment failure definition [1]. There is limited data available about the course of the hematoma volume over time after embolization.

Therefore, we evaluated the treatment response of recurrent chronic subdural hematomas to embolization in comparison to second surgery. Additionally, we demonstrate by means of computed volumetric assessment the course of the hematoma volume over time after embolization and second surgery and analyzed possible patient and hematoma characteristics.

## Methods and materials

### Patient selection

We retrospectively analyzed patients who underwent treatment for recurrent chronic subdural hematoma after initial burr hole trephination from August 2019 to June 2022. We included recurrent hematomas which were either treated by second surgery (conventional group) or by embolization of the middle meningeal artery as stand-alone therapy (embolization group).

Data was obtained from medical reports and cranial CT scans. Data acquisition included the patients’ age, the pre-interventional clinical status (modified Rankin Scale), history of trauma, intake of anticoagulants or antiplatelet drugs before the diagnosis of chronic subdural hematoma and the intake of statins. Furthermore, the total length of hospital stay during treatment for recurrent hematoma was determined.

### Collection of radiological data

Regarding the collection of radiological data, patients’ CT scans were evaluated at different stages of the treatment course: before and after the initial surgery, before the treatment of recurrent hematoma as well as during the follow-up period. Follow-up CT scans were divided into an early and a late CT scan. The early CT scan was conducted within the first two weeks after treatment and the late one from 2 to 8 weeks after treatment.

All CT scans were assessed by two examiners, who measured the volume by the below mentioned software and the midline shift independently to each other. Only if discrepancies in the measurements occurred, the values were discussed between the examiners and were solved by agreement.

From the imaging we collected the midline shift, the presence of a bilateral hematoma and the radiological type according to the modified Nakaguchi Classification with further subtypes in the homogenous groups based on the density of the hematoma (homogenous hypodense, homogenous iso-dense, homogenous hyperdense, trabecular, laminar, gradation, separated) [25]. Furthermore, the volume of the hematoma was measured by the software Visage Imaging VISAGE 7 Version 7.1.15. For all volumetric measurements CT scans with 1 mm slides were used. First the image was transferred into a 3D image. Then automated volumetric assessment in each slide was performed. Each slide was checked by the examiners and was manually corrected if necessary. Total volume was then calculated. The used embolic agent was recorded as well from the radiological reports.

### Outcome assessment

Treatment failure was defined as the necessity for retreatment in case of second recurrence, either with surgery or embolization.

The above-mentioned patient and hematoma characteristics were analyzed for a possible influence on outcome. These factors were analyzed for the whole patient cohort as well as for the two subgroups.

Volumes were compared before and after treatment in the different follow-up scans within each treatment group.

Furthermore, the time between different treatment options and treatment for second recurrence was measured.

### Statistical analysis

Statistical analysis was performed with IBM SPSS-Software Version 27. Categorial variables were tested with the Fisher’s exact test or with the Fisher-Freeman-Halton-Exact test if the variable had more than two expressions. Odds ratios (OR), 95% confidence intervals (CI), and Cramer’s *V* are given for dichotomous variables. Continuous variables were first tested for normal distribution with the Kolmogorov–Smirnov test. Then non-parametric tests such as the Mann–Whitney-*U* test and the Wilcoxon signed-rank test were applied. The continuous variables are given as mean and standard deviation (SD). Maximum, minimum, and median volumes are given as well.

Significance level of < 0,05 was considered statistically significant in two-sided testing.

## Results

### Patient and hematoma characteristics

A total of 50 recurrent hematomas were treated in 42 patients after initial surgery either with embolization or second surgery during the time-period from August 2019 and June 2022. Twenty-three hematomas in 18 patients were treated by embolization of the middle meningeal artery and 27 hematomas in 24 patients were treated by surgery again.

The mean age of all patients was 77.5 years (SD 11). The age of the patients in the embolization group (78.3, SD 11) did not differ from the age of the conventional group (76.4, SD 11.3) (*p* = 0.633). The clinical presentation before treatment for recurrent hematoma was classified according to the modified Rankin Scale (mRS). Three patients had no symptoms, in 18 patients the mRS was 1, in 17 patients 2, in 1 patient 3, in 2 patients 4 and in one patient 5. The symptoms of the patients varied from no symptoms over headache and motor deficit to disturbance of consciousness. The mRS score was higher in the conventional group (*p* < 0.004). All three asymptomatic patients were treated by embolization. Twenty-eight patients took either anticoagulants or antiplatelet drugs before diagnosis. In most cases the medication was discontinued at the time of diagnosis of the hematoma. Nineteen patients were on a statin therapy throughout the treatment course. In 28 patients, a history of trauma was recorded during the stay for the first treatment of the hematoma.

Nineteen hematomas possessed a contralateral hematoma; however, only in 8 cases both hematomas were treated. Bilateral hematomas were more common in the embolization group (*p* = 0.02). According to the modified Nakaguchi Classification, most hematomas appeared trabecular (*n* = 18) or homogenous hypodense (*n* = 17). Homogenous iso-dense (*n* = 7) and gradation types (*n* = 7) as well as one hematoma with the separated type were present. The distribution among the groups did not differ (*p* = 0.343). The mean midline shift in all patients was 4.57 mm (SD 3.74). It differed in the subgroups significantly (conventional group 7.12 mm, SD 3.35 vs. embolization group 2.67 mm, SD 2.76; *p* < 0.001). Mean volume of all recurrent hematomas was 89 ml (SD 44.4). The volume in the different treatment groups did not differ significantly (conventional group 101.7 ml, SD 53.7 vs. embolization group 75.1 ml, SD 27.3; *p* = 0.09). As expected, patients with more severe clinical status and with hematomas with higher volume and midline shift were more likely to be selected for second surgery. Patients with bilateral hematoma were more likely to be selected for embolization.

The embolic agents in the embolization group were Onyx (*n* = 17), Squid (*n* = 5), and PHIL (*n* = 1).

Patients who were readmitted for recurrence had a mean total length of hospital stay of 7.46 days (SD 3.93). In the conventional group the stay was not significantly longer with mean duration of 8.93 days (SD 4.84) compared to 5.45 days (SD 1.97) in the embolization group (*p* = 0.138).

Patient and hematoma characteristics are summarized in Table 1.

**Table 1:** Patient and hematoma characteristics in all patients and in the two subgroups. Outcome and complications are given as well

			All patients	Conventional group	Embolization group	*p* value
Patient characteristics						
	number		42/42	24/42 (57)	18/42 (43)	
	Mean age (years)		77.5 (SD 11)	78.3 (SD 11)	76.4 (SD 11.3)	0.633
	mRS					< 0.004
		0	3/42	0/24	3/18	
		1	18/42	8/24	10/18	
		2	17/42	12/24	5/18	
		3	1/42	1/24	0/18	
		4	2/42	2/24	0/18	
		5	1/42	1/24	0/18	
	Symptoms					
		Headache	12/42	6/24	6/18	
		Motor deficit	15/42	7/24	8/18	
		Gait disturbance	2/42	1/24	1/18	
		Aphasia	5/42	5/24	0/18	
		Cognitive impairment	5/42	3/24	2/18	
		Seizure	2/42	2/24	0/18	
		Reduced vigilance	2/42	2/24	0/18	
	Anticoagulants or antiplatelet drugs before diagnosis		17/42	8/24	09/18	
	Statin therapy		19/42	8/24	09/18	
	History of trauma before first treatment		28/42	16/24	12/18	
Hematoma characteristics						
	number		50/50	27/50	23/50	
	Mean pre-interventional Volume in ml		89 (SD 44.4)	101.7 (SD 53.7)	75.1 (SD 27.3)	0.09
	Mean pre-interventional Midline shift in mm		4.57	7.12	2.67	< 0.001
	Modified Nakaguchi Classification					0.343
		Homogenous hypodense	17/50	11/27	6/23	
		Homogenous isodense	7/50	3/27	4/23	
		Trabecular	18/50	7/27	11/23	
		gradation	7/50	3/27	4/23	
		Separated	1/50	1/27	0/23	
	Presence of bilateral hematoma		19/50	6/27	13/23	0.020
	Embolic agent					
		Onyx			17/23	
		Squid			5/23	
		PHIL			1/23	
Outcome						
	Complications		4/50	2/27	2/23	
		Empyema	2/50	2/27		
		Mild bleeding from a. iliaca externa (without intervention)	1/50		1/23	
	Need for re-treatment	mild ischemia	11/50	8/27	3/23	
	Mean Time in days until re-treatment		15.3	18.5	6.7	0.376
	Efficacy		39/50 (78%)	19/27 (70,3%)	20/23 (87%)	0.189

### Outcome and possible influencing factors

At the first recurrence 23 hematomas were treated with embolization of the middle meningeal artery and 27 hematomas with surgery again.

Eight out of 27 (26,6%) surgically treated recurrent hematomas failed to respond and needed to be treated again as second recurrent hematoma. Three out of twenty-three (13%) of the hematomas treated by embolization needed to be rescued by surgery due to worsening symptoms again in the follow-up course. This leads to an efficacy in recurrent hematomas of 73,4% in surgically treated and of 87% in embolized hematomas. This difference was not statistically significant (*p* = 0.189, Cramer’s V = 0.2, OR 0.356, CI 95% 0.082–1.546) (Table 1). So, embolization seems to be equally effective as surgery in the treatment of recurrent chronic subdural hematomas.

**Fig. 1:**
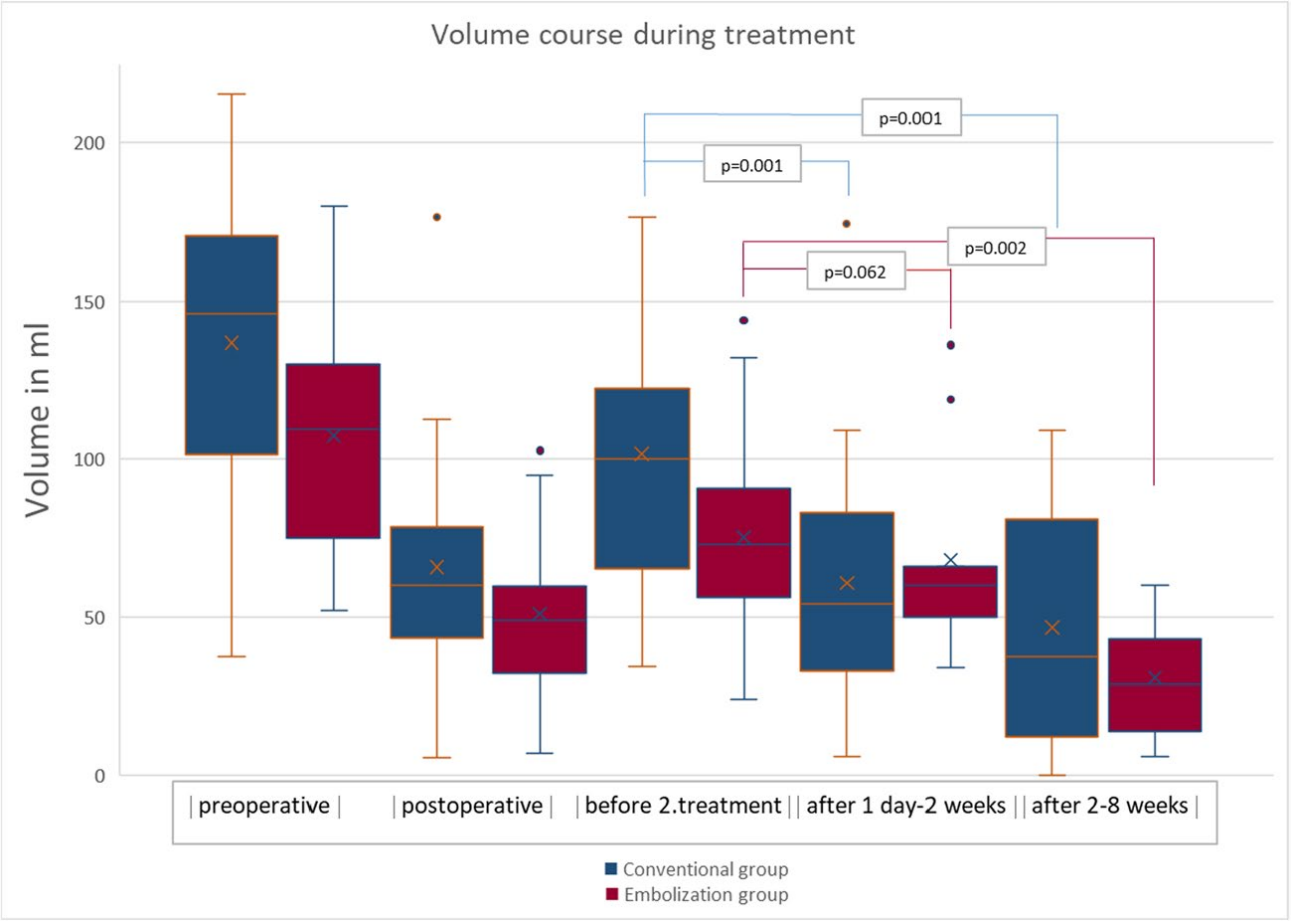
All hematomas were surgically treated at first. Second treatment was either second surgery (blue) or embolization (red). Volume decreased in both groups, but as expected, it was faster in the conventional group

Time between treatment for second recurrence differed between the groups. The mean time in days between second surgery and treatment for second recurrence was 18.5 days and the time between embolization and treatment for second recurrence was only 6.7 days. This is probably due to initial volume decrease and the need for re-collection in surgically treated patients (Fig. 1).

Four complications in the whole patient cohort occurred during the treatment course. In the conventional group, two patients developed a subdural empyema. In the embolization group one patient developed small areas of ischemia, detected in a post-interventional MRI, without residual neurological symptoms. Another patient had a mild bleeding from the a. iliaca externa, however without the need for surgical intervention.

No patient characteristic such as age, the intake of anticoagulants or antiplatelet drugs before the diagnosis of chronic subdural hematoma, the intake of statin nor the history of trauma for the initial hematoma did have a significant impact in all patients or in the subgroups.

The clinical status measured by the modified Rankin Scale did also not influence the outcome. However, none of the three asymptomatic patients treated with embolization required retreatment.

Concerning the influence of radiological characteristics only the pre-interventional/surgical hematoma volume in all patient was higher in failed hematomas (*p* = 0.023). In the subgroups, it was not significant. Neither the presence of a bilateral hematoma nor the radiological type had a significant impact on treatment response.

The different embolic agent did also not influence the need for surgery either.

Outcome assessment with given *p*-values, Cramer’s *V* values and OR with CI are given in Table 2.

**Table 2:** Outcome parameters with clinical and radiological factors

		All patients	Conventional group	Embolization group
Patient characteristics				
	Number of failed hematomas	11/50	8/27	3/23
	Mean age (years)	*p* = 0.8	*p* = 0.291	*p* = 0.1
	mRS	*p* = 0.748	*p* = 0.198	*p* = 0.635
	Anticoagulants or antiplatelet drugs	*p* = 1 Cramer’s V = 0.006 OR 1.03 95% CI 0.19–5.68	*p* = 1 Cramer’s V = 0.006 OR 1.03 95% CI 0.19–5.68	*p* = 0.560 Cramer’s V 0.181 OR = 3 95% CI 0.23–38.88
	Statin therapy	*p* = 1 Cramer’s V = 0.037 OR 1.2 95% CI 0.31–4.61	*p* = 1 Cramer’s V = 0.043 OR 0.83 95% CI 0.15–4.5	*p* = 0.560 Cramer’s V = 0.181 OR 3 95% CI 0.23–38.88
	History of trauma before first treatment	*p* = 0.475 Cramer’s V = 0.128 OR 0.53 95% CI 0.11–2.09	*p* = 0.375 Cramer’s V = 0.229 OR 0.357 95% CI 0.06–2	*p* = 1 Cramer’s V = 0.012 OR 1.08 95% CI 0.08–14.08
Hematoma characteristics				
	pre-interventional/surgical Volume	*p* = 0.023	*p* = 0.066	*p* = 0.265
	mean pre-interventional Midline shift in mm	*p* = 0.116	*p* = 0.364	*p* = 0.898
	Modified Nakaguchi Classification	*p* = 0.226	*p* = 0.424	*p* = 0.077
	Presence of bilateral hematoma	*p* = 1 Cramer’s V = 0.039 OR = 0.82 95% CI 0.21–3.28	*p* = 0.633 Cramer’s V = 0.199 OR = 0.31 95% CI 0.03–3.11	*p* = 0.229 Cramer’s V = 0.340 OR 1.3 95% CI 0.97–1.75
	Embolic agent			*p* = 0.127

### Volume course in the different treatment groups

During the first treatment all hematomas were surgically treated.

Initial mean volume of 121.4 ml (SD 43.2) decreased to 58.1 ml (SD 33.2) in the postoperative CT scan (*p* < 0.0001). Then, during the follow-up course mean volume increased significantly again to 89 ml (SD 44.7) in all patients (*p* < 0.0001). Before treatment of recurrent hematoma, volume was non-significantly higher in the conventional group (101.7 ml, SD 53.7) than in the embolization group (75.1 ml, SD 27.3) (*p* = 0.09).

In the postoperative phase, most patients received an early follow-up CT scan (1 day to 2 weeks) and a late follow-up CT scan (2–8 weeks).

In the conventional group, volume decreased significantly already in the first follow-up CT scan from 101.7 (SD 53.7) to 60.7 ml (SD 40.3) (*p* = 0.001) and dropped further in the later follow-up scan to 46.6 ml (SD 37.1) (*p* = 0.001).

As expected, volume decrease in the embolization group was slower. In the embolization group, the volume did decrease from 75.1 ml (SD 27.3), not significant though, to 68 ml (SD 31.4) in the first scan (*p* = 0.062). However, in the late scan significant volume reduction to 30.8 ml (SD 17.1) could be observed (*p* = 0.002).

As expected, volume decrease occurs faster in the conventional group, however, embolization leads to sufficient volume reduction in recurrent hematomas later in the follow-up course.

Volume courses are summarized in Fig. 1 and Table 3.

**Table 3:** Volume parameters in the conventional and embolization group over time

		Conventional group	Embolization group
Volume in ml	Mean		
	Median		
	Minimum		
	Maximum		
	Preoperative	136.9	107.3
		146	109.3
		37.3	52
		215.5	180
	Postoperative	65.7	50.9
		59.9	48.9
		5.7	6.9
		176.4	102.7
	Before second treatment	101.7	75.1
		100.2	73
		34.1	24
		269.3	144
	After 1 day – 2 weeks	60.7	68
		54	60
		6	34
		174.4	136.1
	After 2–8 weeks	46.6	30.8
		37.2	28.5
		0	6
		109	60

## Discussion

In our study, we demonstrate that embolization of the middle meningeal artery is one option for recurrent chronic subdural hematomas especially in patients with mild symptoms who can tolerate slow volume reduction. Embolization of the middle meningeal artery is able to reduce volume of recollected recurrent hematomas (Fig. 1). In our cohort, the pre-interventional volume and midline shift in the embolization were lower and the symptoms were milder than in the conventional group. With this distribution of these parameters, we achieved an equally good treatment response. Probably these parameters must be considered in the decision-making which treatment option to choose.

In small case series, the efficacy of the embolization on recurrent chronic subdural hematomas were analyzed. Thomas et al. presented six out of 7 recurrent hematomas, which were successfully treated by embolization. Kan demonstrated in a small case series a better treatment response of recurrent chronic subdural hematoma to embolization than to second burr hole (*p* = 0.008). In a meta-analysis by Nia et al., rescue embolization for recurrent hematomas did not differ in terms of treatment response compared to primary or adjunctive embolization [18].

In our study, treatment response was not different in the two groups (*p* = 0.189). Volume reduction was obviously faster in the conventional group than in the embolization group. Therefore, surgery is still the only useful treatment option for patients with severe symptoms who need quick volume reduction for symptom relieve. However, in patients with mild symptoms, embolization is a sufficient treatment option. Patients with more severe clinical status, higher volume and midline shift were treated by second surgery more likely. Advantages of the embolization are the shorter length of hospital stay and that treatment failure might be detected earlier in the follow-up course than after surgery.

Despite the addition of medical therapy and embolization to surgery in the treatment for primary hematomas, recurrent hematomas will still appear and need to be treated if the patient is symptomatic. Three of our patients in the embolization group were asymptomatic. Ethical aspects for treatment of asymptomatic should be considered. Every patient should be individually assessed and be informed about different treatment strategies, including watch and wait, and their possible complications. Treatment of asymptomatic patients might be indicated in cases who need therapy with anticoagulative or antiplatelet drugs, e.g., for treatment of ischemic heart disease. It is not necessary to discontinue these medications during the treatment course with the embolization of the middle meningeal artery [15].

Despite the obvious fact, that surgery reduces volume quicker, Housley et al. compared hematoma volume change after treatment with surgery or embolization as a sole therapy in primary hematomas. They noticed a significant faster resorption rate in the first 24 h (*p* < 0.001) and in the interval 3–12 weeks (*p* < 0.001) in the surgery group. Later in the follow-up course the resolution rates were similar [7].

In our study, neither the age nor the midline shift did show an influence on the outcome. These findings were observed in other studies as well [11, 20, 26]. Pre-interventional/surgical volume in the whole patient cohort was the only significant factor, but it was not significant in the subgroups. Pre-interventional volume did not influence the outcome in the embolization group, which is line with other studies [11, 20]. Furthermore, the radiological type was also not an influencing factor in our patient cohort. The early and intermediate stage hematomas (homogenous hyper and iso-dense, separated, gradation, laminar) did show a trend, though not significant, to better resorption in the study by Khorasanizadeh et al. [11].

No significant difference on volume reduction and recurrence after embolization was observed between patients using and not using antithrombotic agents in the postoperative phase after surgery in other studies [15, 23]. There should not be an influence of these agents after embolization since all the blood supply to the vulnerable vasculature of the membrane should be vanished. In our study, it did not influence the outcome. Statin therapy showed a reduced risk of recurrence in surgically treated patients, however not in embolized patients [6, 19]. In our study, it did not influence the treatment response.

The published studies and ongoing trials all use different embolic agents and even coils. There are some recommendations to use liquid embolic agents rather than particle agents due to better penetration into the small vasculature [5]. In this study, Squid, Phil and Onyx were used. We could not detect an influence of these agents on treatment response. This is in line with other studies, which did not find a significant difference between the materials used to embolize the artery [10, 12, 23].

In summary, embolization is as effective as surgery in the treatment for recurrent chronic subdural hematomas, despite slower volume reduction. Especially in patients with mild symptoms, who can tolerate slow volume reduction, embolization should be considered to avoid recurrent surgery.

### Strengths and limitations

The main limitations of the study are its retrospective design with a different number and scheduled follow-up CT scans as well as the rather low number of cases.

The main strength of the study is the availability of the hematoma volume and a control group. The measurement by software is more precise than the maximal width and provides more information about the disease.

## Conclusions

In our study, we demonstrate that the embolization of the middle meningeal artery is an effective treatment option for recurrent chronic subdural hematoma. However, volume reduction is slower in comparison to second surgery. Therefore, we recommend the embolization in recurrent hematomas if symptoms are mild and slower resorption can be tolerated. Larger prospective studies are necessary to evaluate the treatment response and limitations of different treatment options for recurrent chronic subdural hematomas.

## Data Availability

The authors confirm that the data supporting the findings of this study are available within the article.
